# Supplementary material for: Radiosensitizing Effect of PARP Inhibition on Chondrosarcoma and Chondrocyte Cells Is Dependent on Radiation LET
Source: Biomolecules. 2024 Aug 27;14(9):1071. doi: 10.3390/biom14091071 (PMC11429578; doi:10.3390/biom14091071)

Supplementary data S1

ns: non significant
*: significant (p<0,05)
**: significant (p<0,01)
***: significant (p<0,001)

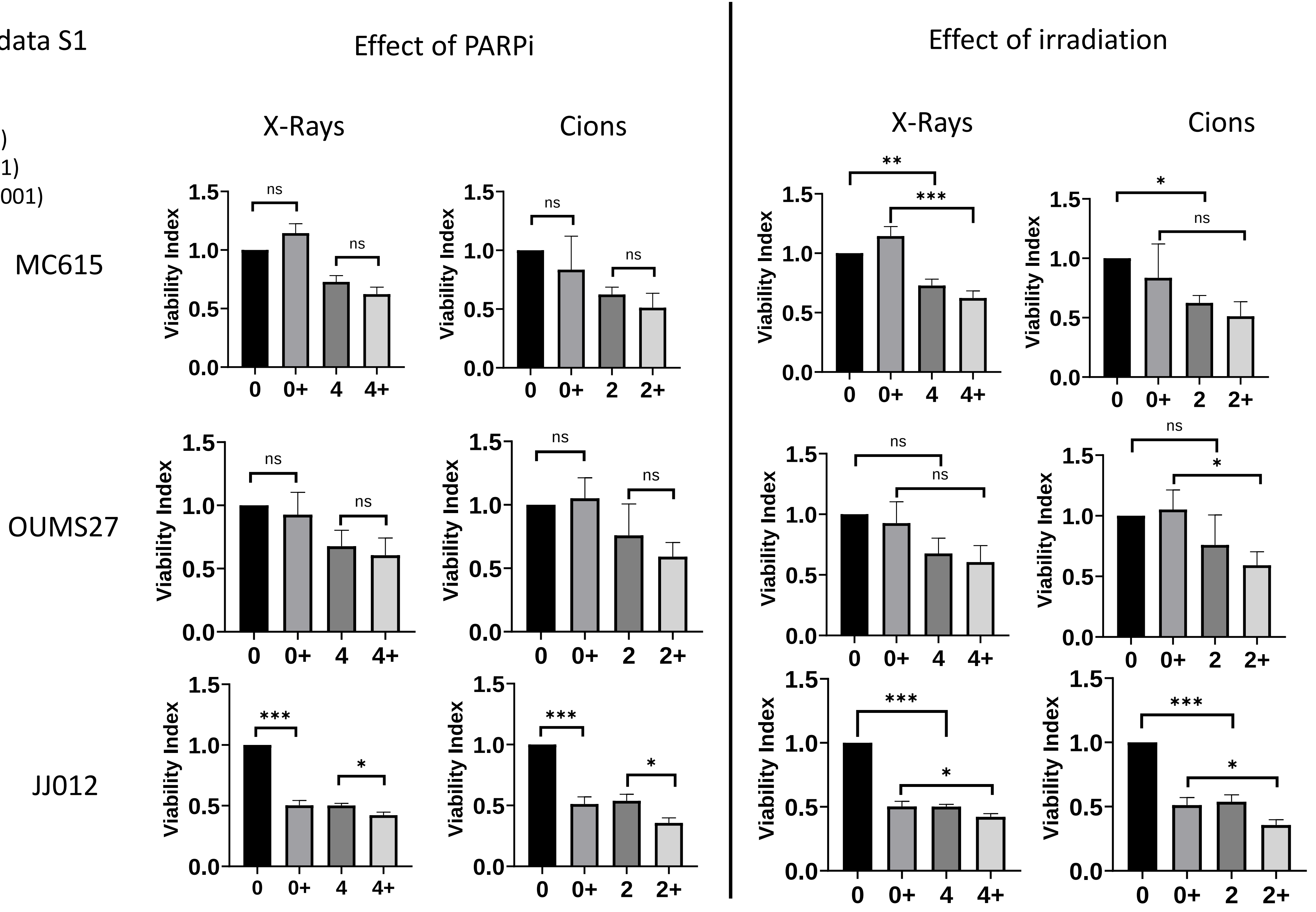

Supplement: Supplementary file 1 [file biomolecules-14-01071-s001.zip › Supplementary data S1.pdf]
