# Supplementary material for: Radiosensitizing Effect of PARP Inhibition on Chondrosarcoma and Chondrocyte Cells Is Dependent on Radiation LET
Source: Biomolecules. 2024 Aug 27;14(9):1071. doi: 10.3390/biom14091071 (PMC11429578; doi:10.3390/biom14091071)
Supplement: Supplementary file 1 [file biomolecules-14-01071-s001.zip › Supplementary data S2.pdf]

Oums27 RX

PARP1
110kDa

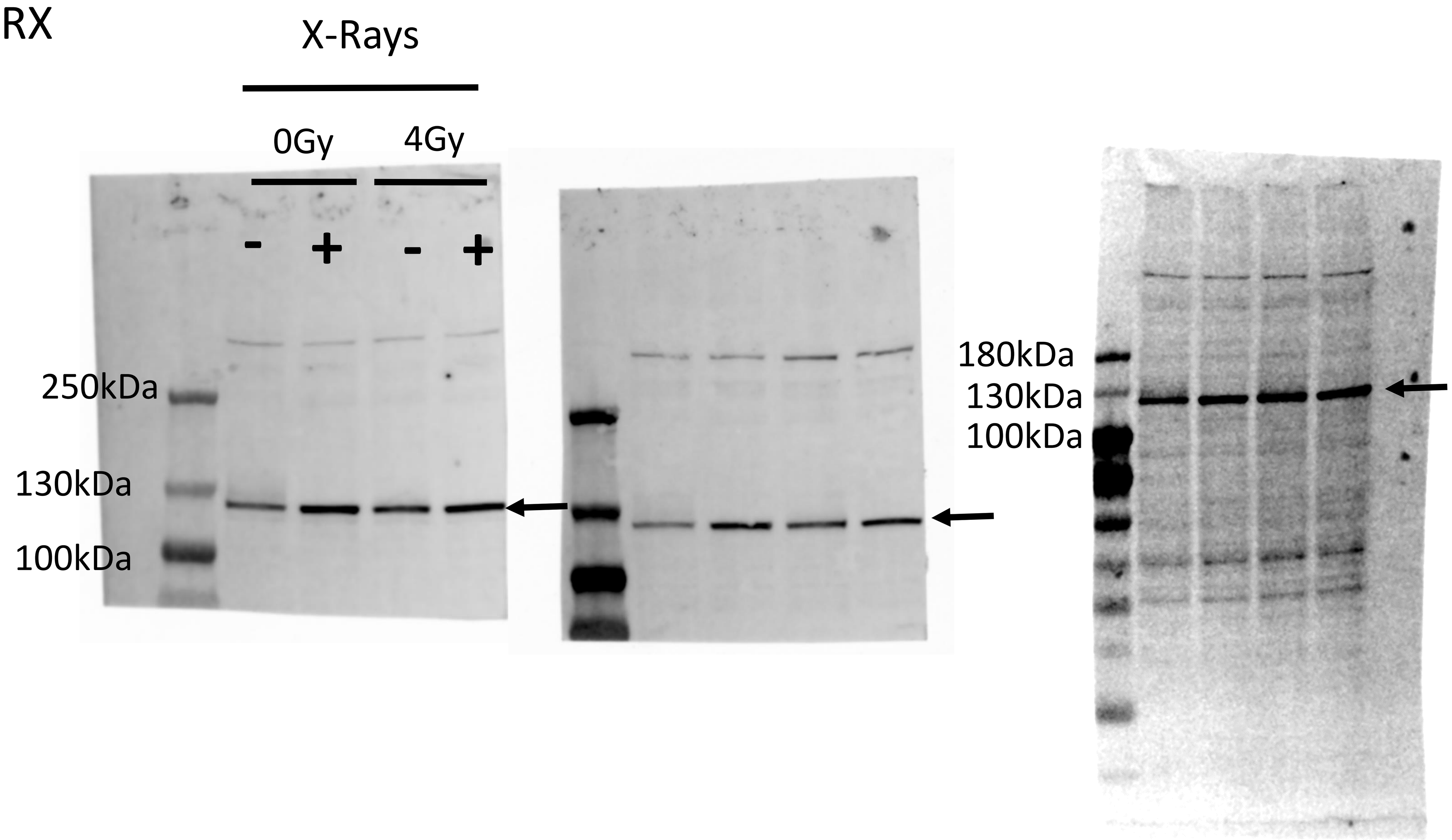

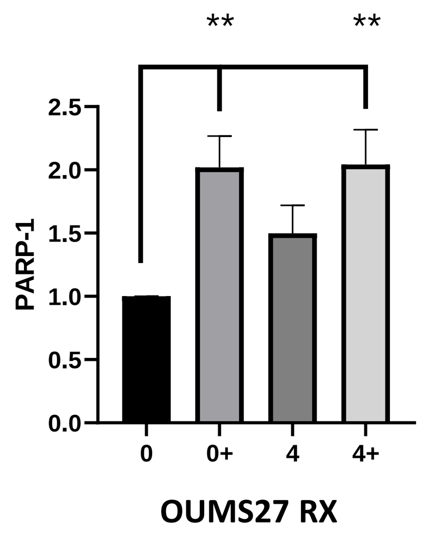

0 =/= 4
0=/= 4+

pATM
350kDa

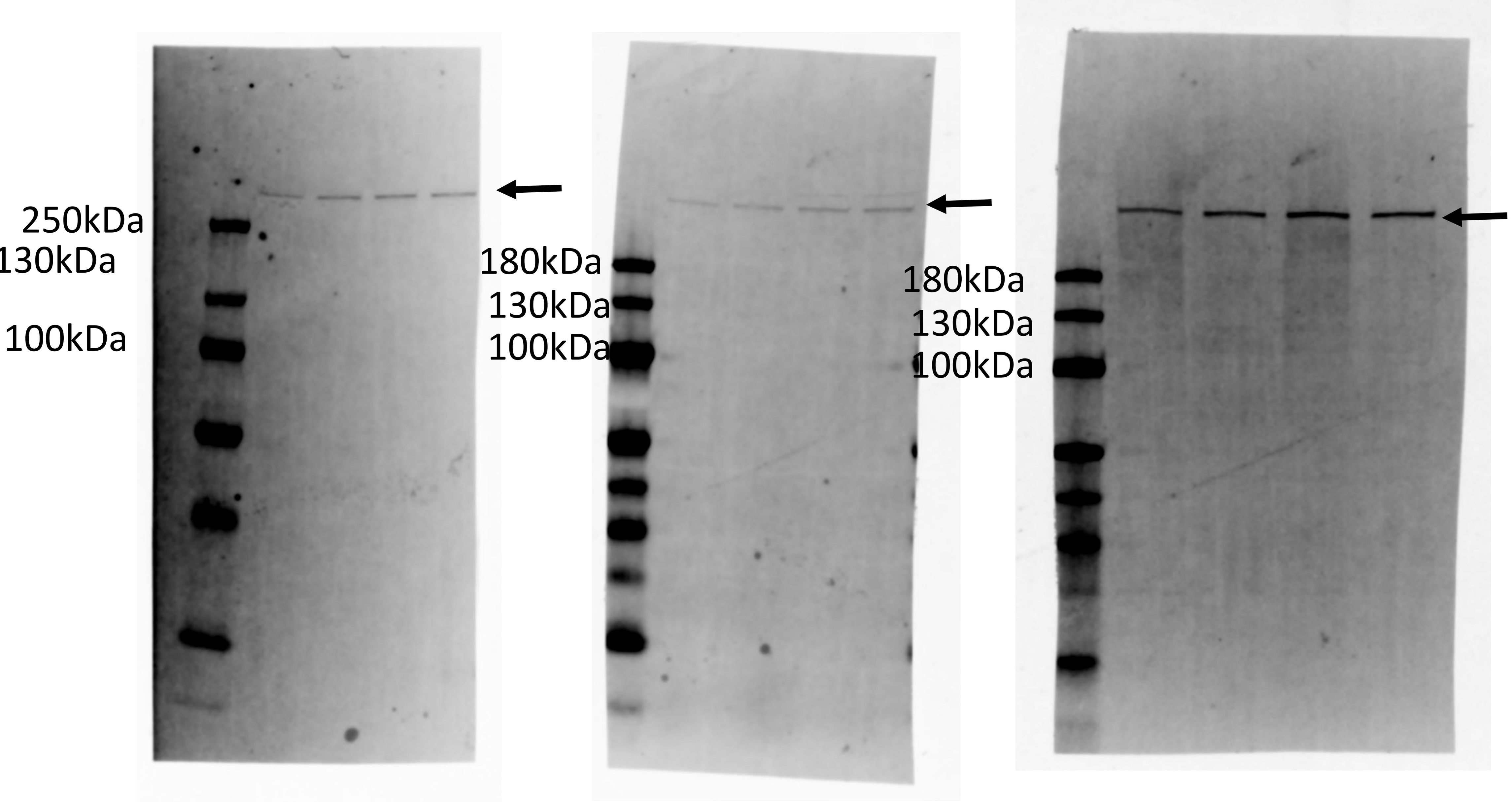

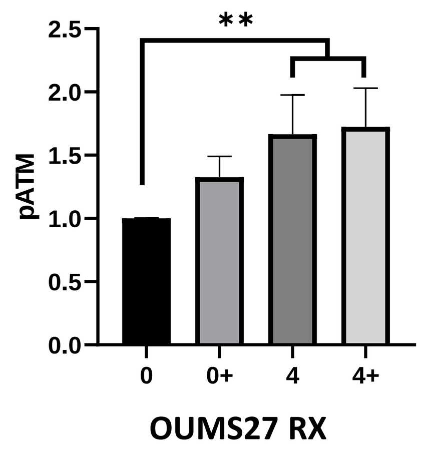

0 =/= 4
0=/= 4+

pDNAPK

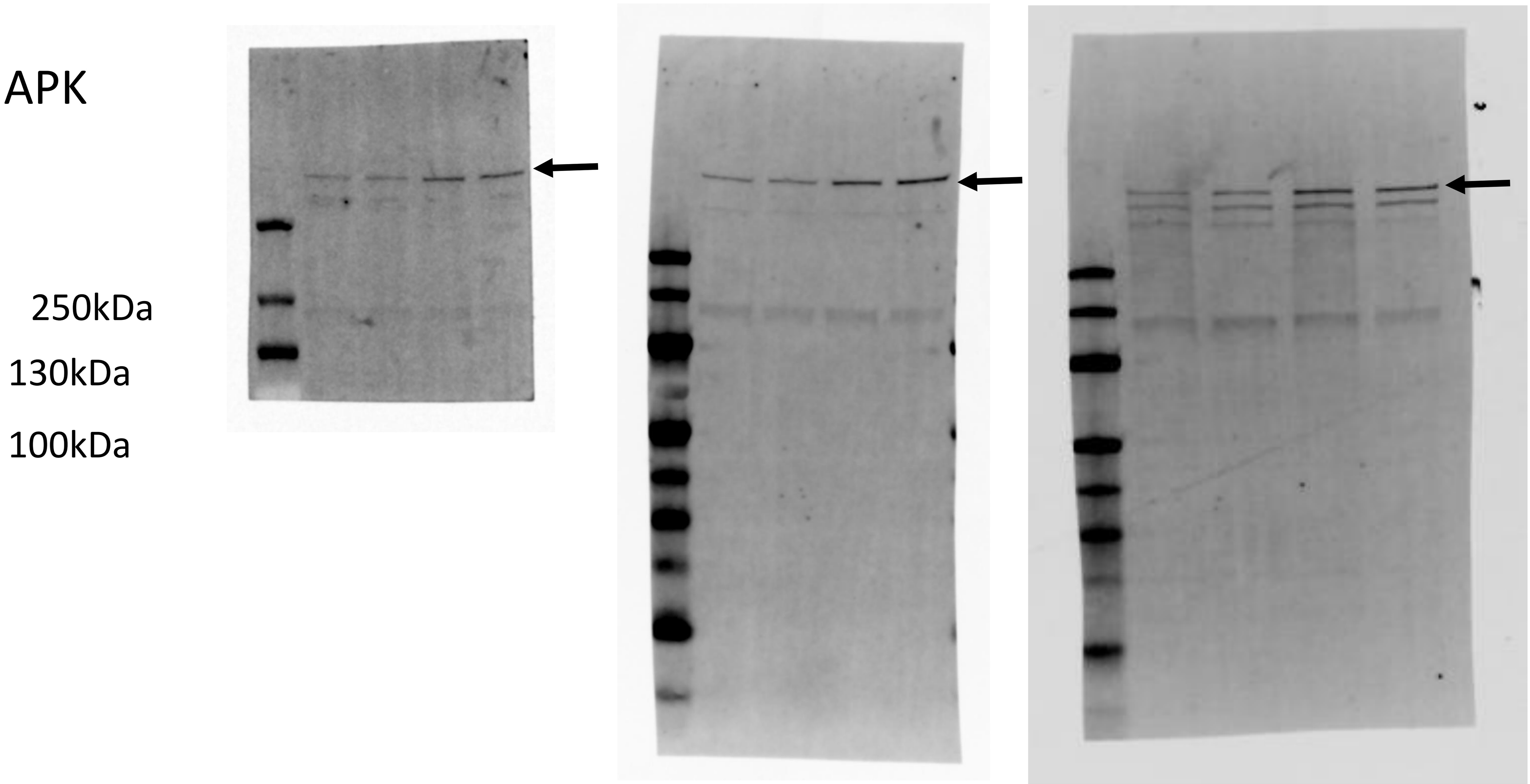

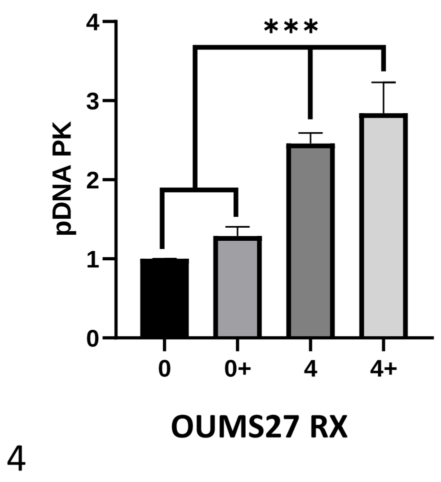

0 =/= 4
0=/= 4+
0+ =/= 4
0+ =/= 4+

pBRCA1

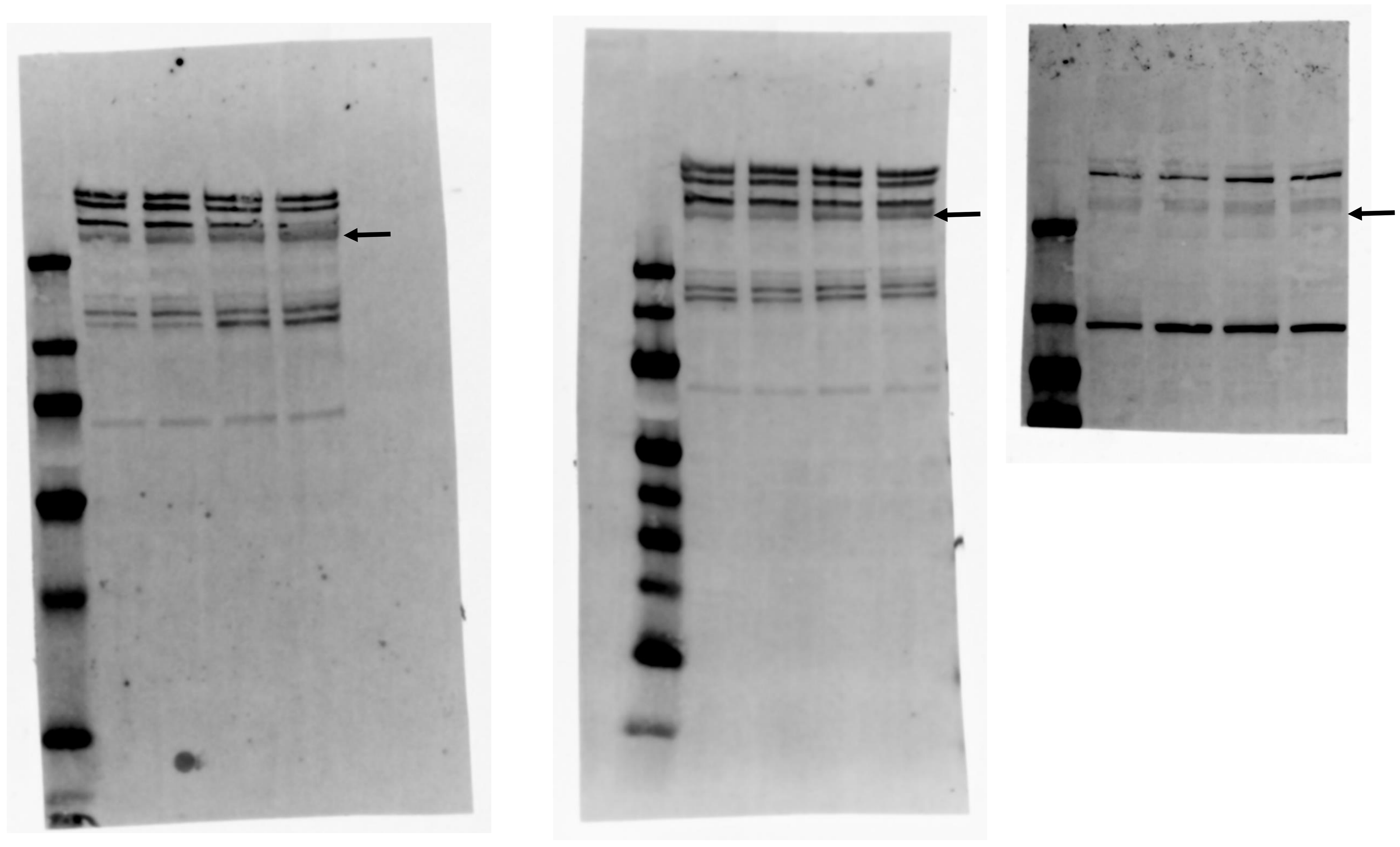

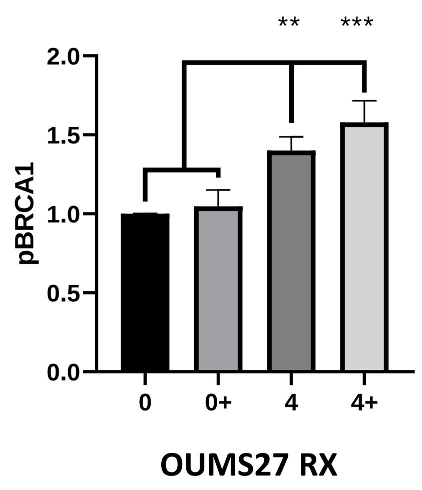

0 =/= 4
0=/= 4+
0+ =/= 4
0+ =/= 4+

0 = 0+
4 = 4+

Oums27 Cions

Cions

0Gy 2Gy

- + - +

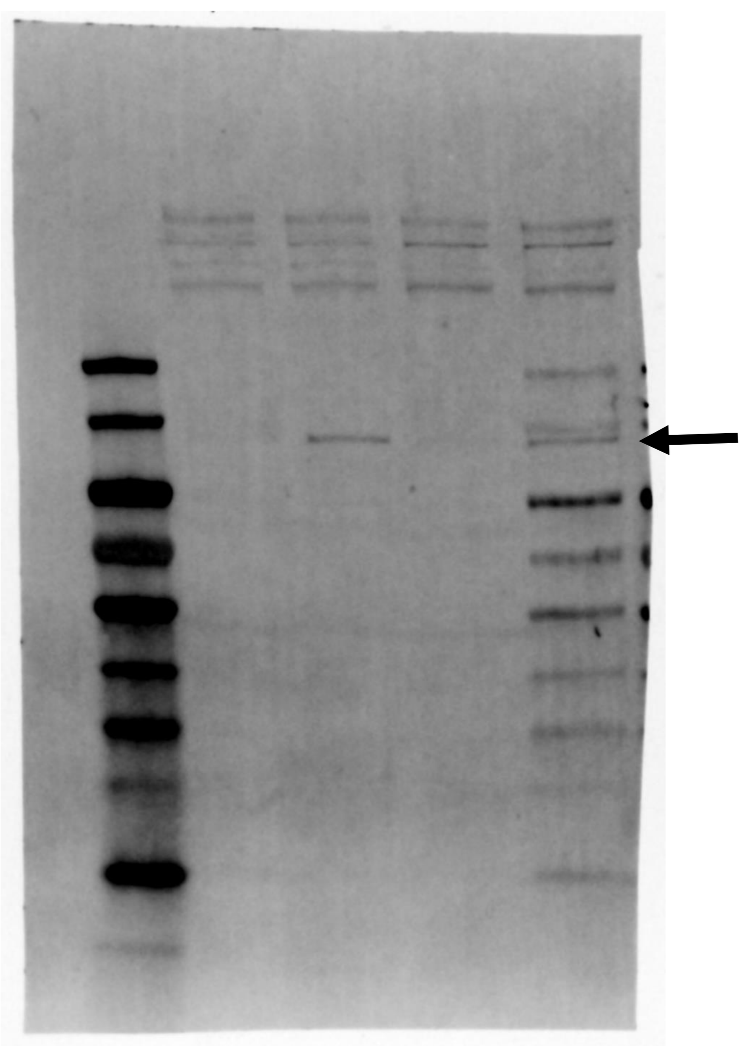

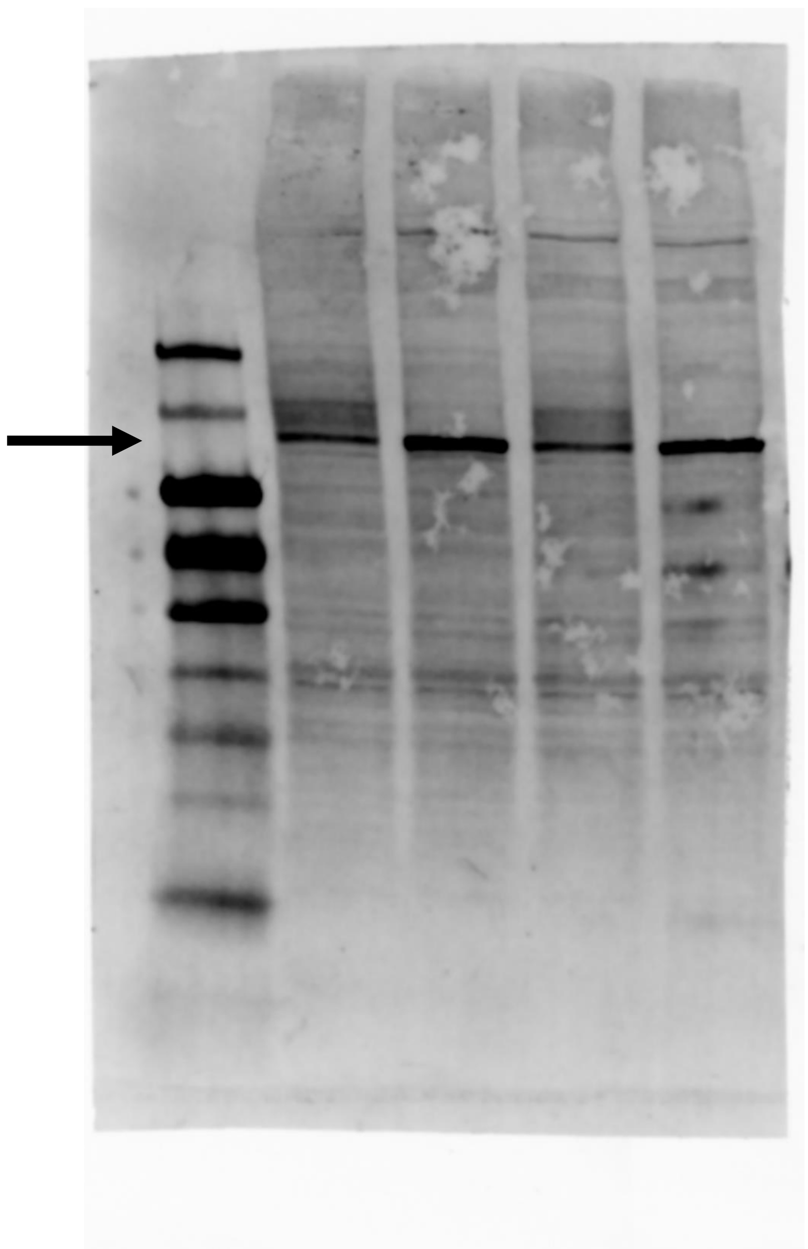

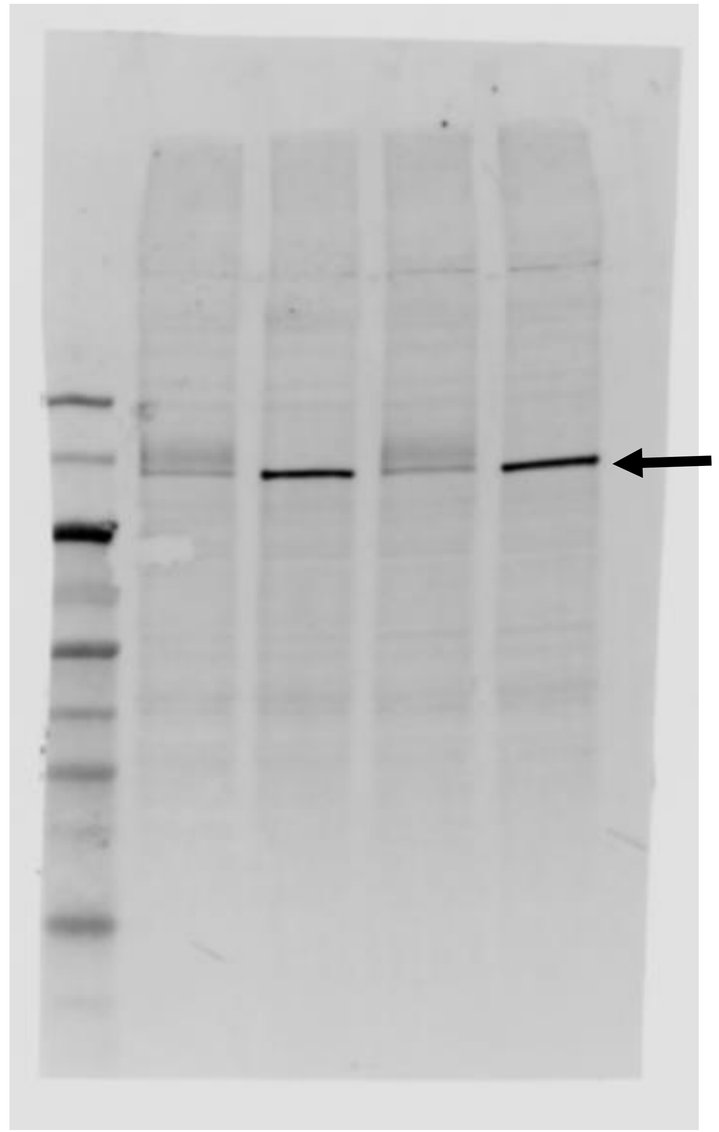

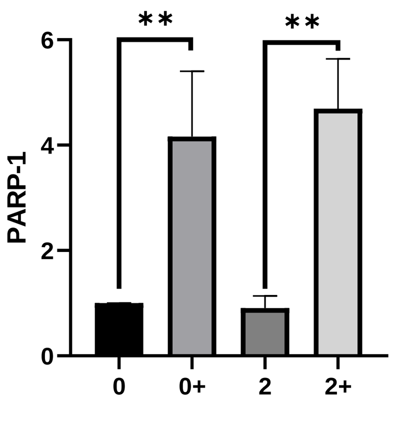

Différence entre :
0 et 0+
2 et 2+
(aussi entre 0 et 2+, …

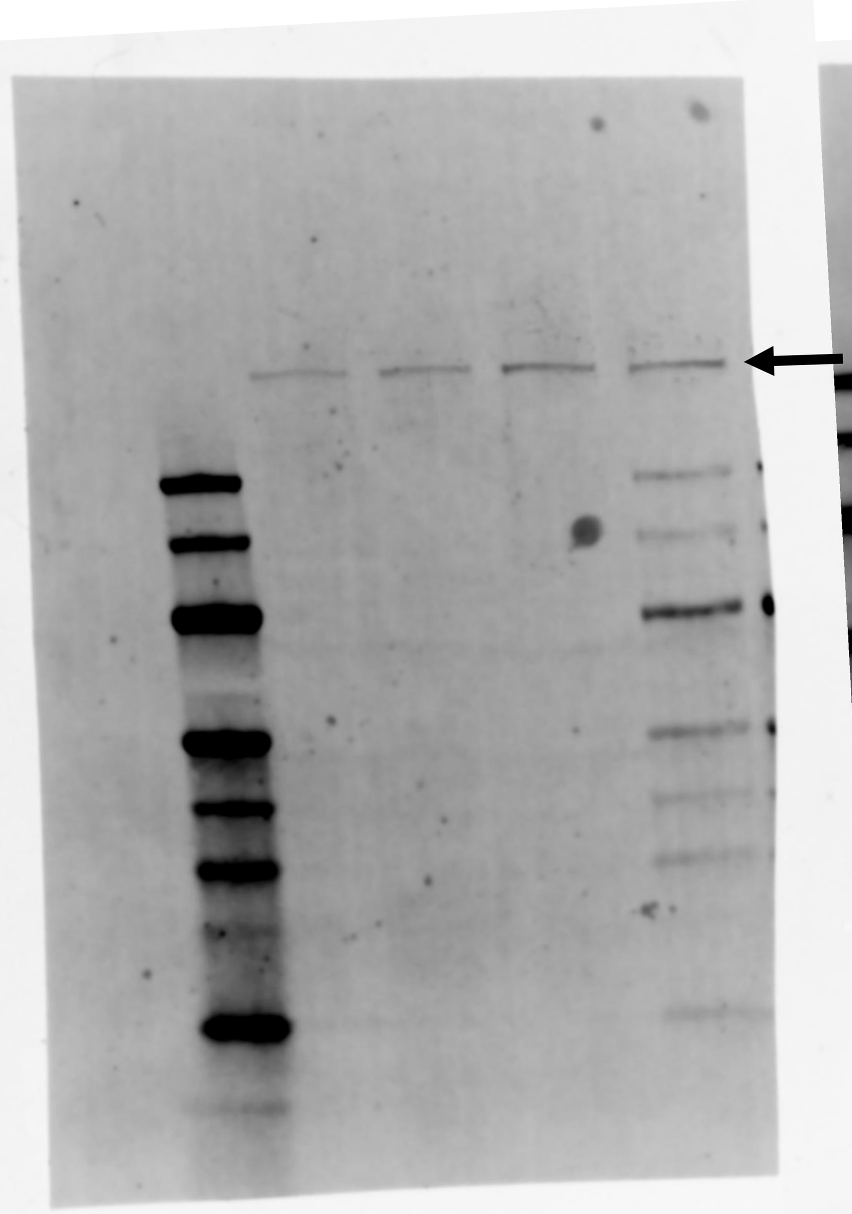

Cions

0Gy 2Gy

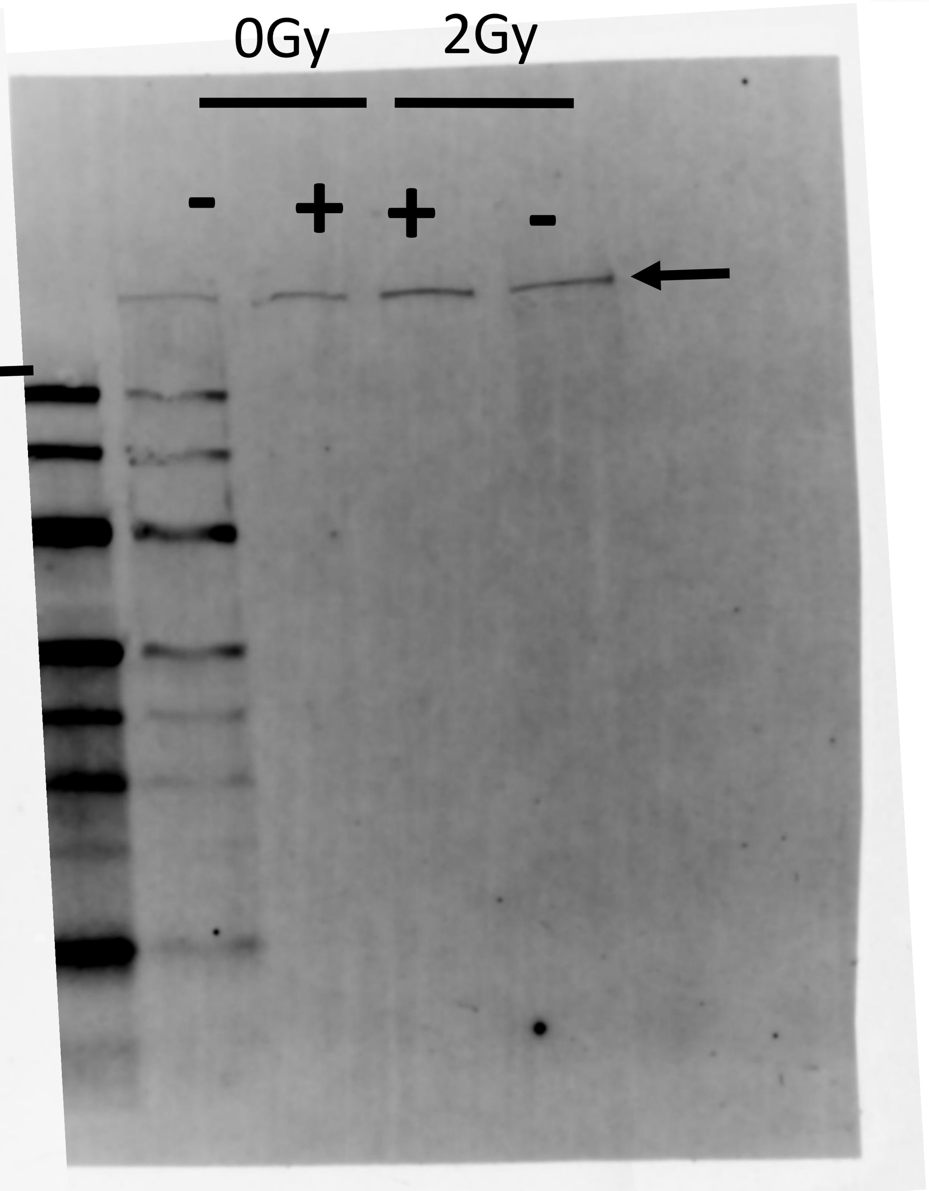

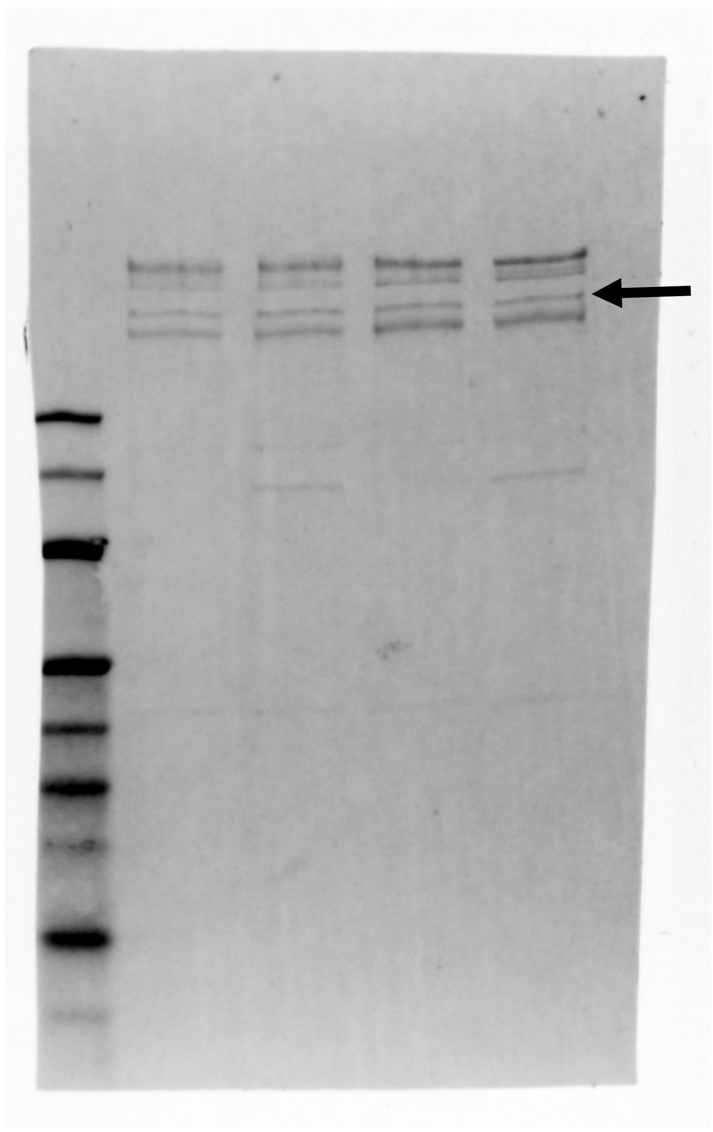

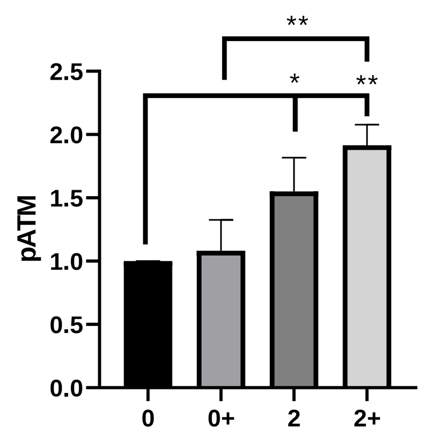

Différence entre :
0=/= 2 et 2+
0+ =/= 2+

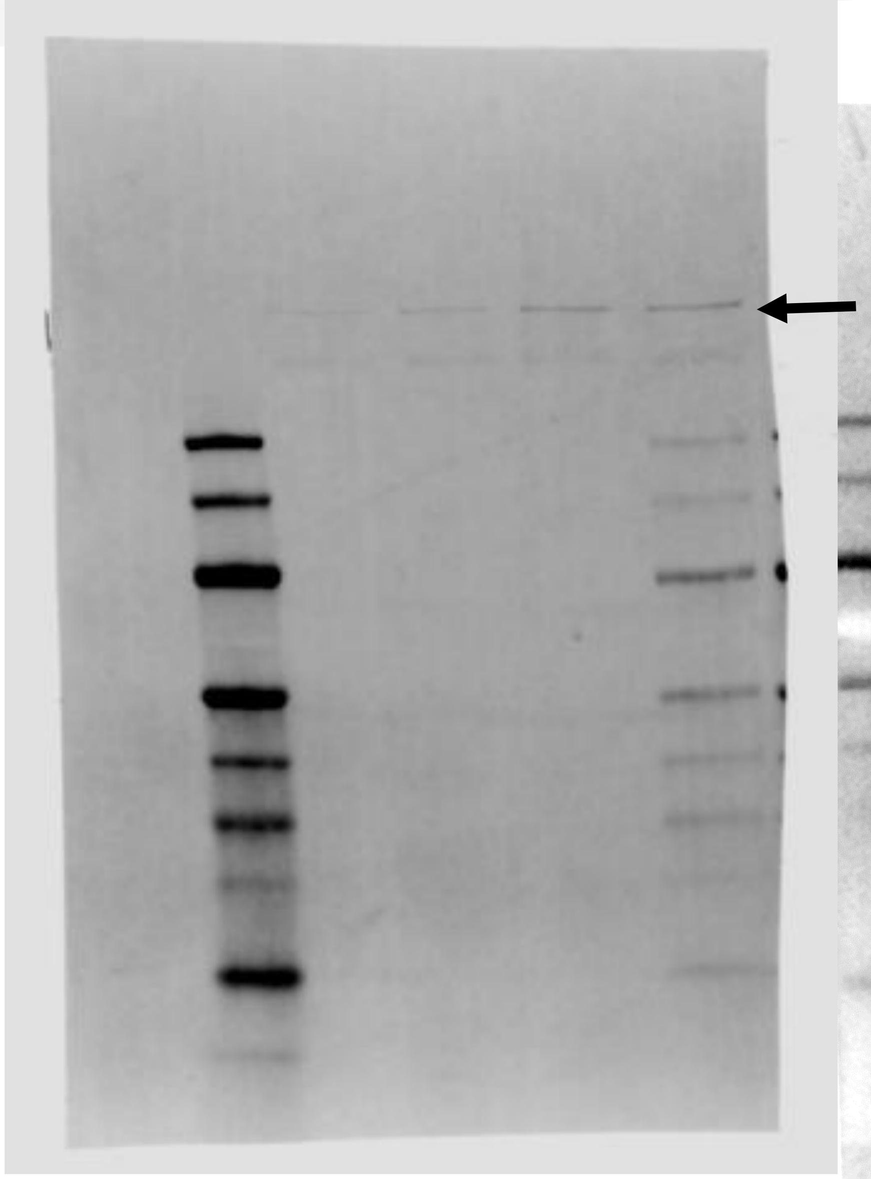

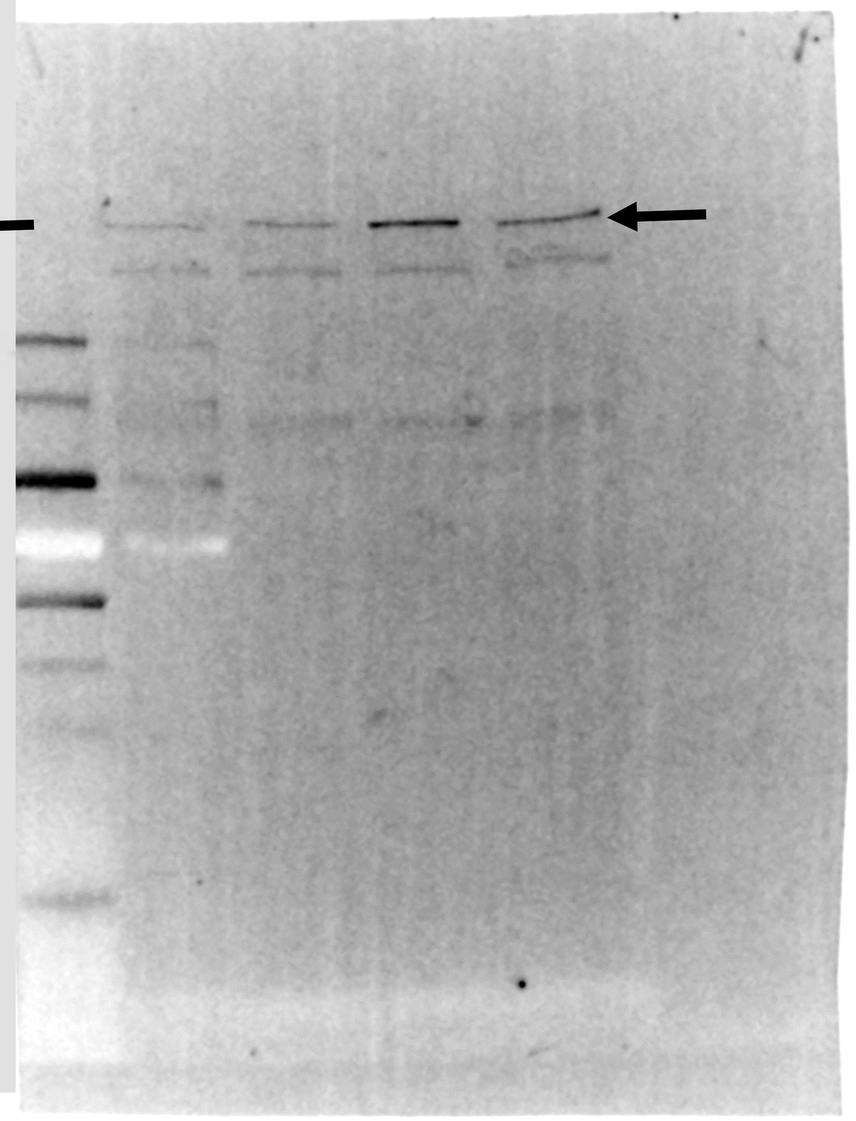

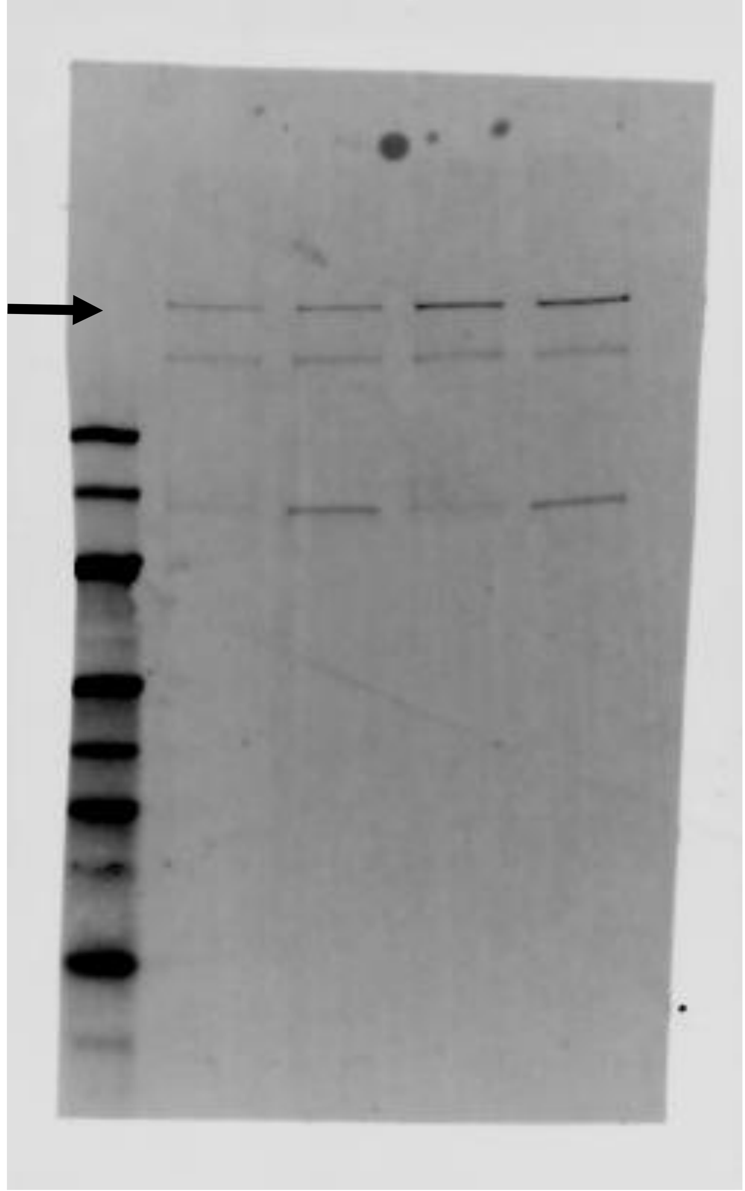

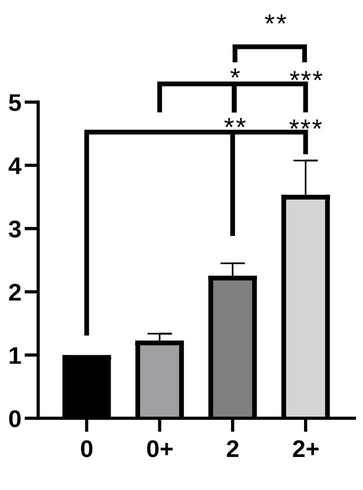

Tout différent sauf 0 = 0+
Sur Graphe :
0 =/= 2 : **
0 =/= 2+ : ****
2 =/= 2+ **

RCA1

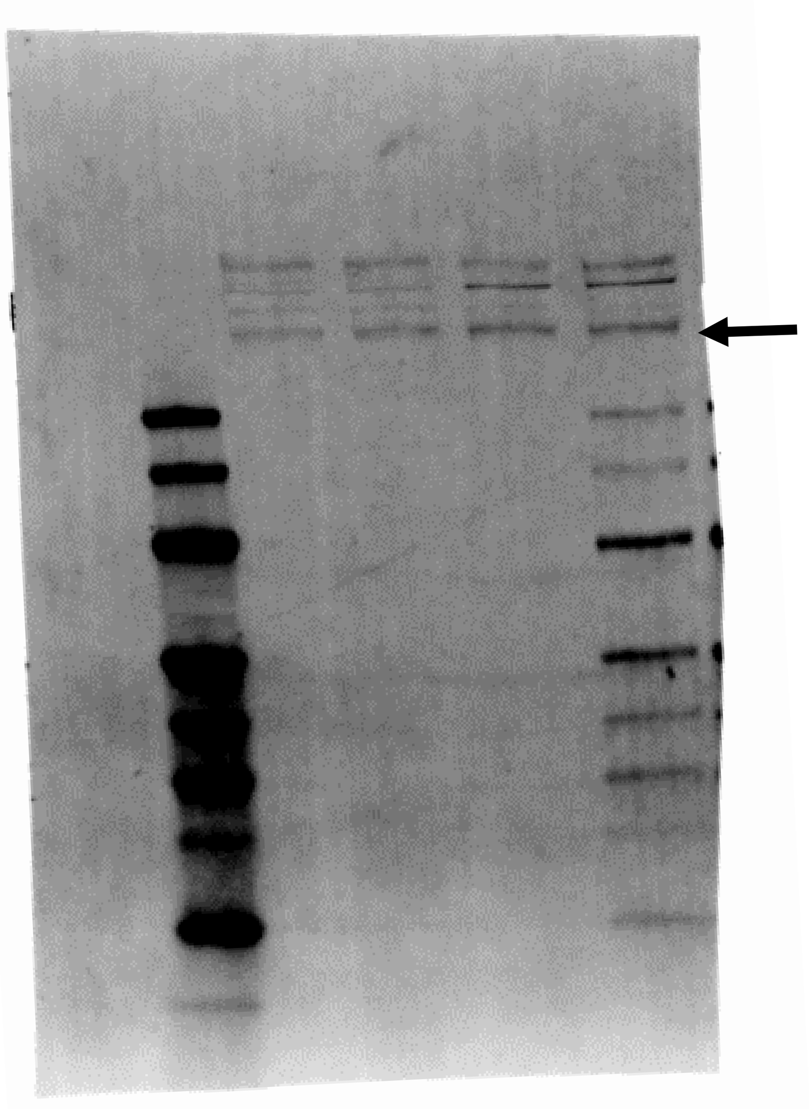

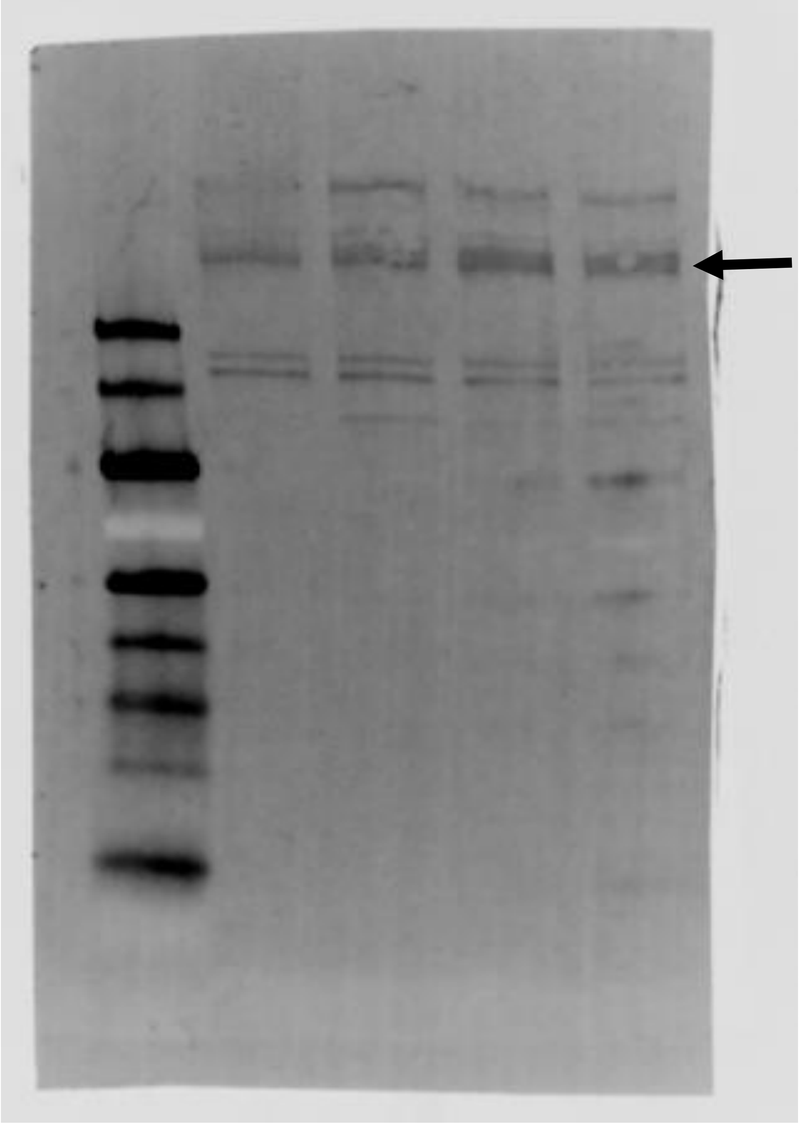

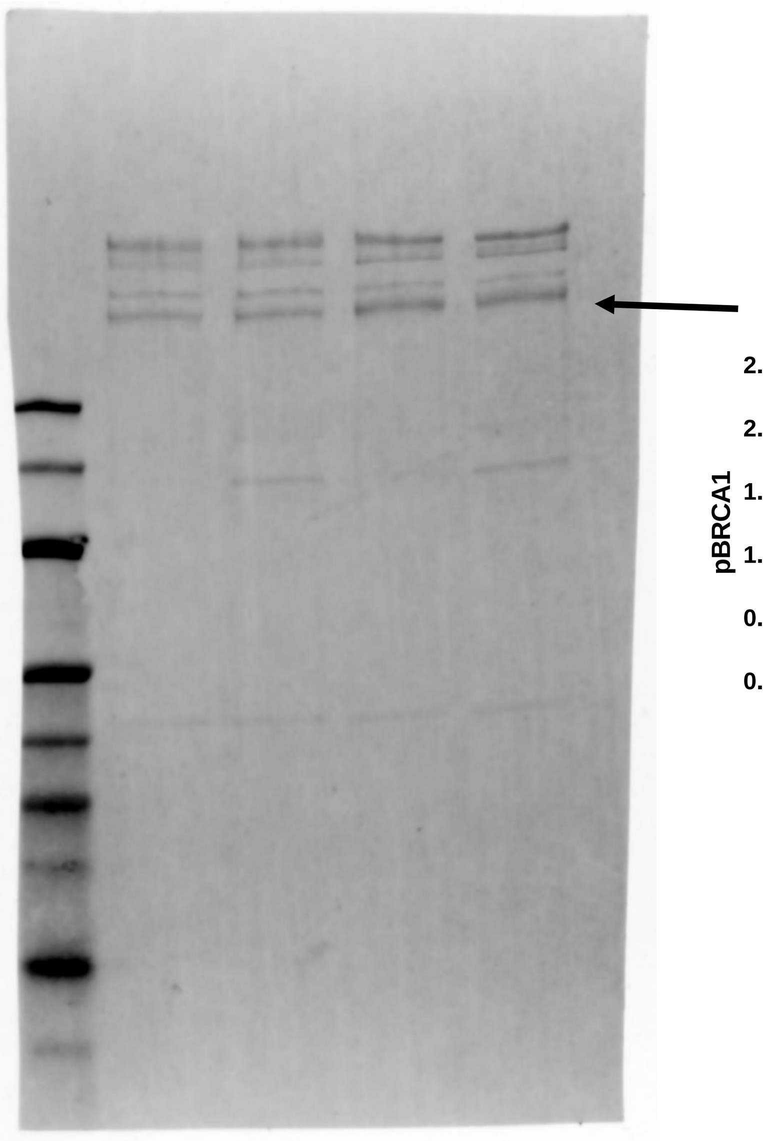

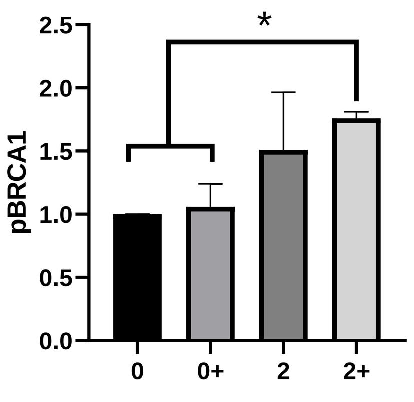

0 =/= 2+
0+ =/= 2+

JJ012 RX

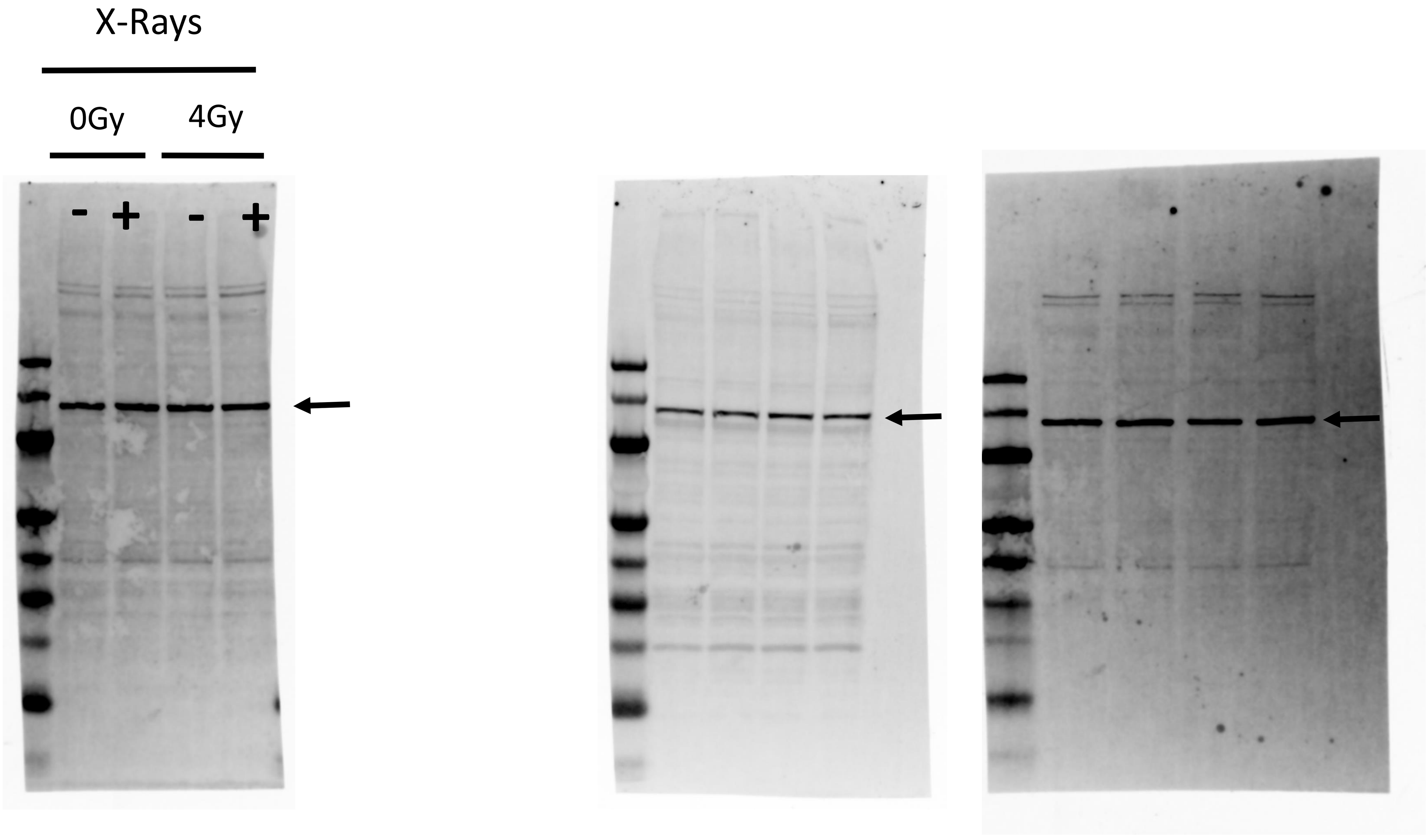

PARP1

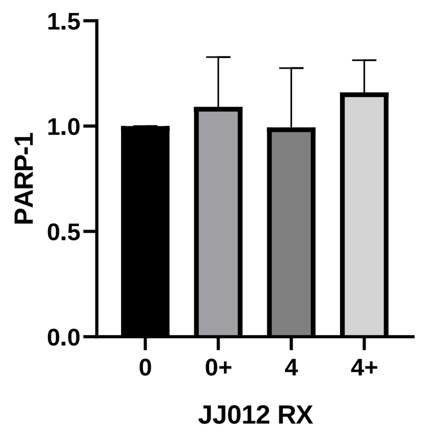

Pas de différence

pATM

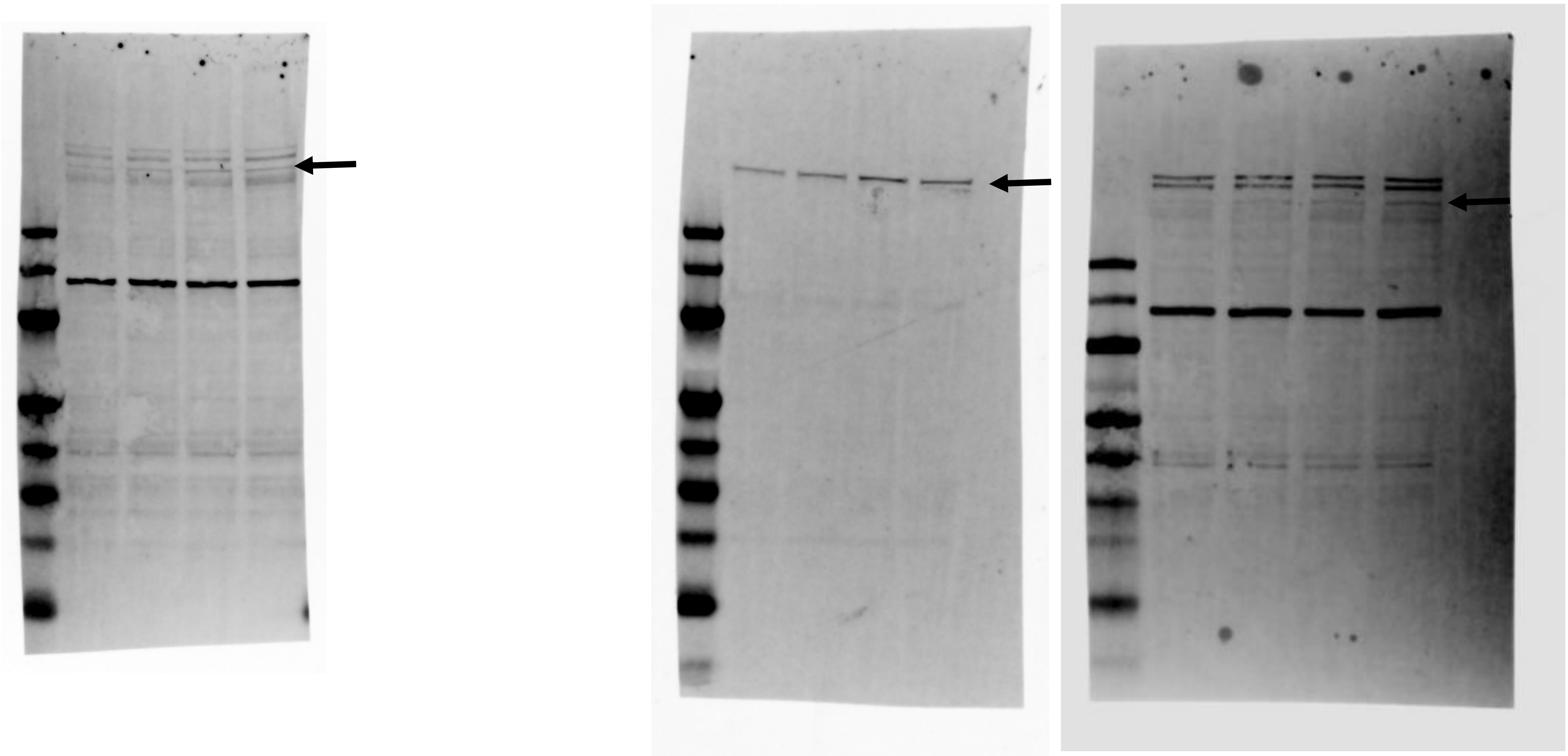

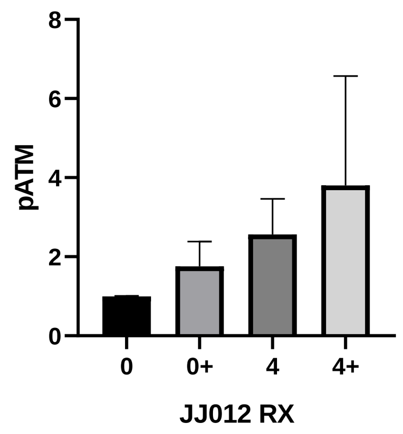

Pas de différence mais grande variabil

pDNAPK

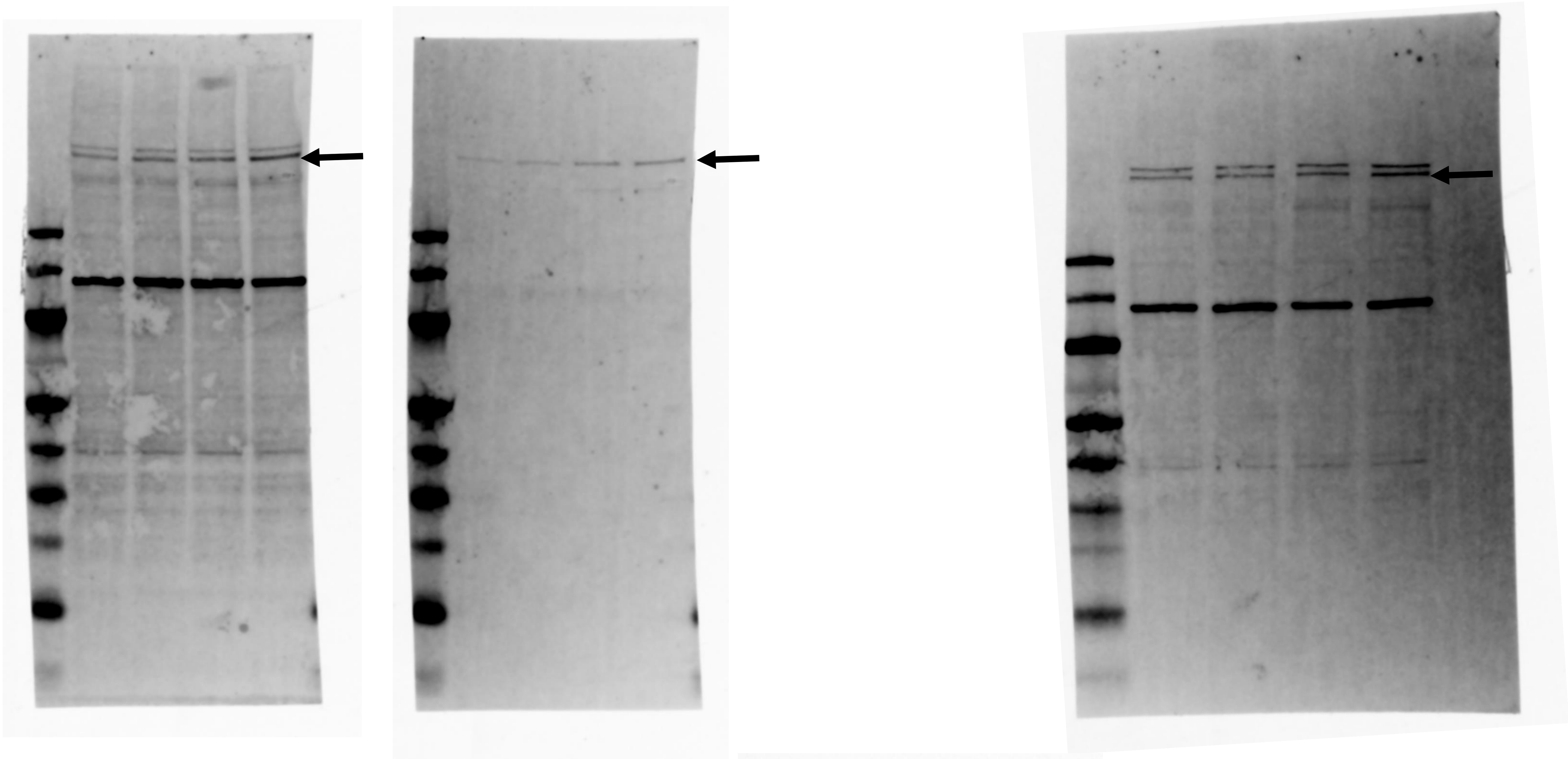

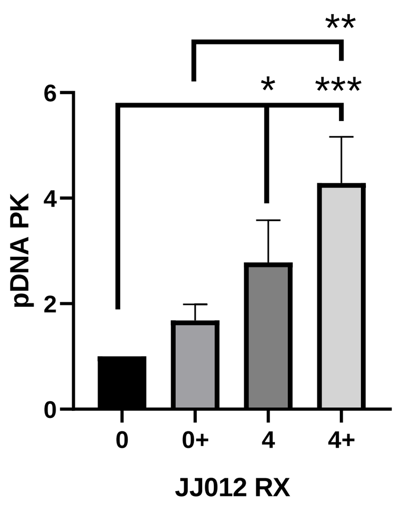

0 =/= 4 et 4+
0+ =/= 4+

pBRCA1

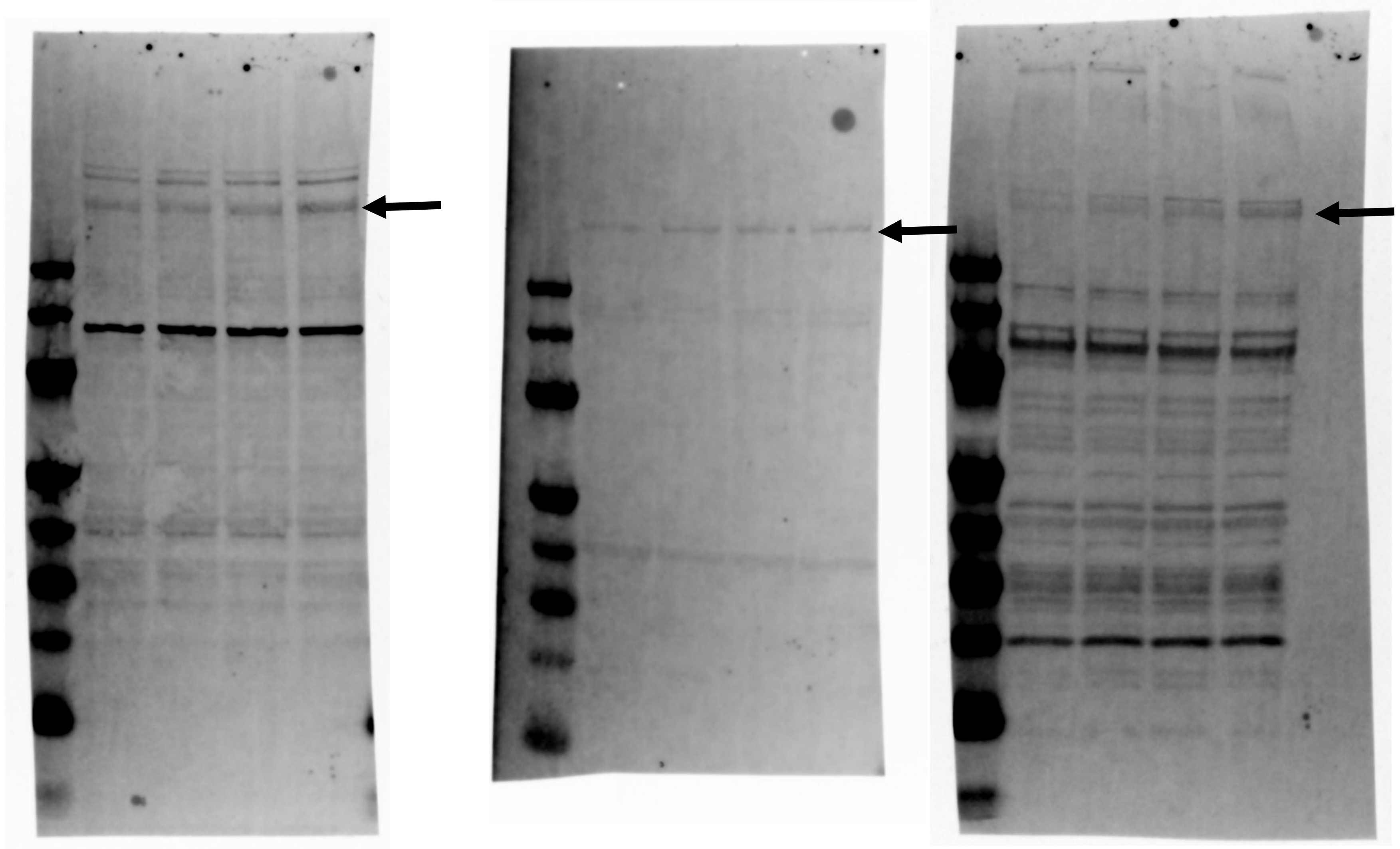

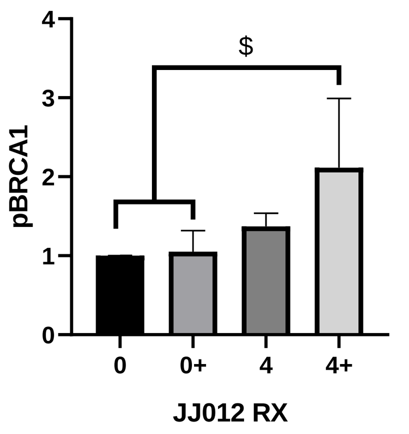

Diff 0 vs 4+ = 0,07
0+ vs 4+ = 0,08

À améliorer en retirant l'acrylamide)

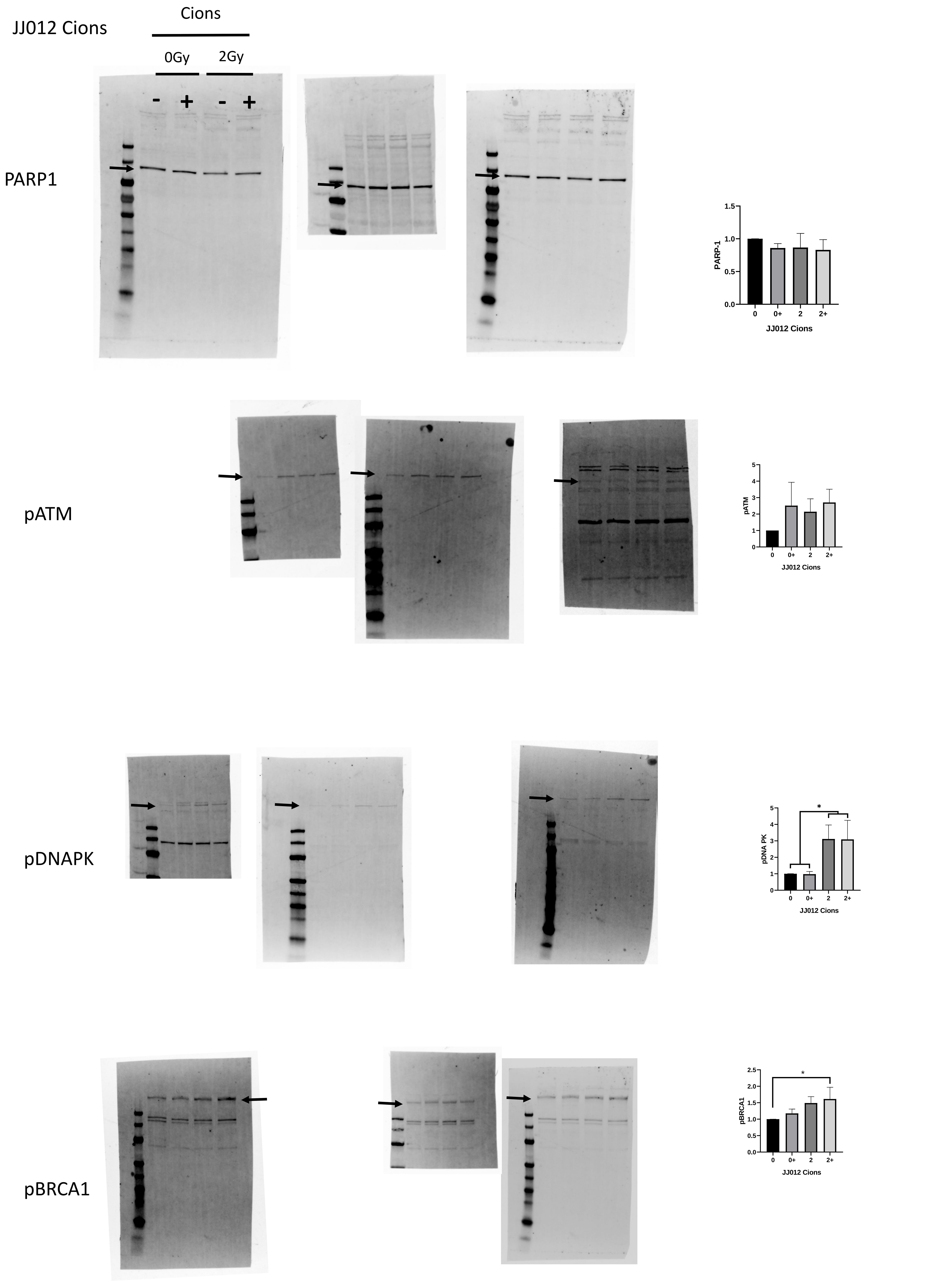
JJ012 Cions
Cions
0Gy
2Gy
- + - +
PARP1
pATM
pDNAPK
pBRCA1
PARP-1
1.5
1.0
0.5
0.0
0 0+ 2 2+
JJ012 Cions
pATM
5
4
3
2
1
0
0 0+ 2 2+
JJ012 Cions
pDNA PK
*
5
4
3
2
1
0
0 0+ 2 2+
JJ012 Cions
pBRCA1
*
2.5
2.0
1.5
1.0
0.5
0.0
0 0+ 2 2+
JJ012 Cions
